# *Pistia stratiotes* L. Biochar for Sorptive Removal of Aqueous Inorganic Nitrogen

**DOI:** 10.3390/ma17153858

**Published:** 2024-08-04

**Authors:** Eunice O. Babatunde, Ranjit Gurav, Sangchul S. Hwang

**Affiliations:** 1 Ingram School of Engineering, Texas State University, San Marcos, TX 78666, USA; moh21@txstate.edu; 2 Sustainability Cluster, School of Advanced Engineering, University of Petroleum & Energy Studies, Dehradun 248007, Uttarakhand, India; ranjitg.gurav@ddn.upes.ac.in

**Keywords:** biochar, nitrate nitrogen, surface chemistry, water lettuce, adsorption

## Abstract

Biochar has proven effective in the remediation of excess nitrogen from soil and water. Excess nitrogen from agricultural fields ends up in aquatic systems and leads to reduced water quality and the proliferation of invasive species. This study aimed to assess the efficiency of chemically surface-modified biochar produced from invasive *Pistia stratiotes* L. for the adsorption of inorganic nitrogen (NH_4_^+^ and NO_3_^−^). Biochar structure was investigated using scanning electron microscopy, energy-dispersive X-ray analysis, X-ray photoelectron spectroscopy, Fourier-transform infrared spectroscopy, and inductively coupled plasma mass spectrometry. The results from adsorption experiments indicate that NH_4_^+^ removal was optimal (0.8–1.3 mg N g^−1^) at near-neutral pH levels (6.0–7.5), while NO_3_^−^ removal was optimal (0.4–0.8 mg N g^−1^) under acidic pH conditions (4.8–6.5) using the modified biochar. These findings highlight the significance of solution pH, biochar morphology, and surface chemistry in influencing the adsorption of NH_4_^+^ and NO_3_^−^. However, further studies are necessary to assess the potential oxidative transformation of NH_4_^+^ to NO_3_^−^ by biochar, which might have contributed to the reduction in NH_4_^+^ in the aqueous phase.

## 1. Introduction

The pollution of soil, air, and water continues to rise as a result of increased consumption, manufacturing, and industrial processes, emphasizing the critical need for effective environmental remediation solutions. As a result, there is increasing demand for sustainable and environmentally friendly pollution management technologies. Scientists are actively investigating and developing safe, natural, and cost-effective alternatives. Biochar (BC), a potential material for environmental remediation, may be synthesized from waste items, making it both sustainable and economically viable. In recent years, BC has been actively studied for its possible applications in environmental remediation [1]. One notable plant in this context is *Pistia stratiotes* L. (water lettuce), an invasive species that spreads quickly in nutrient-rich rivers [2]. *Pistia stratiotes* is a free-floating, lightweight, perennial monocotyledon plant recognizable by its soft, furry, thick, green leaves resembling a fan-like rose with hairy roots. *Pistia stratiotes* is characterized by a rapid reproduction rate. Plant population can double in less than 3 weeks under favorable temperature and nutrient conditions [3]. The *Pistia stratiotes* plant has been used extensively in research to remove toxic substances such as metals and inorganic compounds from water sources [4]. However, its invasive nature and tendency to outcompete native aquatic species provide considerable ecological concerns. Hence, the improper disposal of plants can lead to a spread in the growth of the plant. To address these concerns, transforming *Pistia stratiotes* into BC has two advantages: it leverages the plant’s remediation capacity while limiting its spread. This technique not only stops *Pistia stratiotes* proliferation but also provides a low-cost and ecologically beneficial remediation product [5]. Some researchers have used plants from phytoremediation and rhizofiltration studies to produce BC for applications in water and soil remediation. Some other studies have applied the resultant BC for resource recovery and soil enhancement in agricultural fields [6,7,8,9,10]. Studies have shown that BC adsorption efficiency can be increased when the material is physically or chemically modified before use [11]. BC can also be regenerated to help with resource recovery efforts and for cost-effectiveness.

BC is a solid material derived from the thermal decomposition of waste biomass (plant, clay, bones, sludge, etc.) in oxygen-free conditions and is rich in carbon [10,12,13]. Due to the wide range of suitable feedstock for BC production, the feedstock material is easily accessible, thus making BC cheaper than alternative environmental remediation materials [2,14,15]. BC is also produced at lower temperatures than activated carbon, and as such, less energy is required to achieve the desired material characteristics [16]. Some characteristics that make BC useful for sorption processes include its developed pore structure, high carbon content, oxygen-containing surface functional groups, cation exchange capacity, and high surface area [17,18]. The physical properties, chemical properties, and elemental concentrations of BC are closely related to the nature of the feedstock used and the pyrolysis temperature. These have been proven to affect BC surface affinity and sorption efficiency [10,17].

Major sources of nitrogen imbalance in the environment are fertilizers and concentrated animal waste from agricultural fields [19,20]. Studies have estimated that only about 50% of the fertilizer applied to farmlands is used by plants. The other portion is transported into water bodies and the atmosphere. The stability and structure of inorganic nitrogen pose a greater environmental risk, as is the case with most other inorganic contaminants. An elevated concentration of ammonia nitrogen (NH_3_ and NH_4_^+^) contributes to eutrophication, decreases oxygen levels [21], impairs internal organs, and disrupts metabolic processes in aquatic organisms [20,22]. The ingestion of nitrate (NO_3_^−^) through drinking water, with the subsequent conversion to nitrite within the body, leads to methemoglobinemia in infants, stomach cancer in adults, and various health issues in livestock [23]. The emission of nitrogen oxides such as nitrogen dioxide, nitric oxide, and nitrous oxide into the atmosphere results in acid rain [24] and ozone depletion [25], respectively. Furthermore, nitrous oxide is a significant greenhouse gas [25]. Excess inorganic nitrogen compounds in waterbodies have also been linked to the overgrowth of invasive aquatic species. As a result of the potential human and environmental health risks attached to the presence of excess inorganic nitrogen, efficient and cost-effective methods for the remediation, recovery, and transformation of inorganic nitrogen compounds should be developed.

The application of BC to remediate inorganic nitrogen from agricultural fields and wastewater treatment plants has gained significant interest in the past few years [26,27,28]. Numerous studies have used BC to individually or simultaneously adsorb nitrogen (NH_4_^+^, NO_3_^−^) and phosphorus (PO_4_^3−^) from water [12,28,29,30,31]. NH_4_^+^ sorption by BC derived from diverse lignocellulosic feedstocks at various temperatures has been previously assessed through batch equilibration methods. For instance, BC produced from bamboo, orange peels, and corn stover has shown effective adsorption and interaction with NH_3_ [22,32,33,34,35,36], while activated carbon has demonstrated catalyst capabilities for the wet-air oxidation of ammonia [37]. Several studies have reported a reduction in soil NH_4_^−^ concentration following the application of BC [33,38]. Furthermore, various hypotheses have been proposed to explain the decrease in nitrous oxide emissions upon BC application. Notably, the capacity for NH_4_^+^ sorption consistently rises as the pyrolysis temperature decreases when comparing BC produced from the same feedstock. However, when considering different studies, it is observed that BC derived from similar feedstocks at comparable temperatures may not consistently demonstrate similar sorption behaviors.

A few studies have prepared BC from *Pistia stratiotes* and applied the material for contaminant removal via adsorption. A study by Zhang et al. [5] involved the co-pyrolysis of *Pistia stratiotes* and sewage sludge for the adsorption of nitrogen and phosphorus from wastewater. Subsequently, the nutrient-rich BC was used as a slow-release fertilizer for crop cultivation. The feedstock was chemically pretreated before pyrolysis at 500 °C with a retention time of 1.5 h. Li et al. [39] used *Pistia stratiotes* in the development of magnetic porous BC. The study involved a two-step process to first produce hydrochar at 240 °C for 4 h followed by chemical activation with KOH before pyrolysis at 800 °C for 2 h. After pyrolysis, the BC was washed with acid before saturation with iron salts. The resulting material was used for the adsorption of diethyl phthalate in aqueous medium. The studies highlighted showed good sorption potential for the contaminants targeted. Despite these promising findings, there is still a considerable research gap in properly understanding and maximizing the application of *Pistia stratiotes*-derived BC for environmental applications. There is a need for more studies that examine the potential of *Pistia stratiotes* BC as the sole adsorbent with simple activation or surface modification processes. Additionally, more studies can contribute to the pool of characterization data that exists on *Pistia stratiotes* BC, which would show its applicability for the removal of non-metals. This study aims to (1) investigate the usability of BC prepared from *Pistia stratiotes* L. biomass for removing inorganic nitrogen from water and (2) to enhance the sorptive removal of inorganic nitrogen (NH_4_^+^ and NO_3_^−^) using post-pyrolysis chemical modifications of BC.

## 2. Materials and Methods

### 2.1. Materials and Reagents

The *Pistia stratiotes* plants (leaves and roots) used in this study were harvested from the San Marcos River in Texas, USA (N 29°50′58.3505″, W 97°51′25.3404″). The harvested samples were field washed and left to air dry for 5 days. Subsequently, they were oven dried at 70 °C for 24 h before being milled and passed through a 2 mm sieve to homogenize the particle size. The milled samples were stored in airtight high-density polyethylene bottles at room temperature and were utilized within six months of preparation. Analytical-grade reagents were purchased from Fisher Scientific (Hampton, NH, USA). Deionized water (18 MΩ) was used to prepare all solutions.

### 2.2. Biochar Preparation

The milled biomass (≤2 mm particle sizes) was dried and weighed in batches for pyrolysis. BC was prepared by heating it at 500 °C for 2 h in a nitrogen-purged compact split-tube furnace (MTI Corporation OTF-1200X-S-NT-LD, Richmond, CA, USA) using a quartz boat (115 mm L × 70 mm W × 30 mm H) inside the furnace, ensuring a controlled environment. Initially, the nitrogen gas flow rate was set at 1500 mL min^−1^ for the first 30 min. Afterward, the purge rate was reduced to 40 mL min^−1^. The furnace was initially heated at a rate of 10 °C min^−1^ until it reached a temperature of 400 °C, after which it was further increased to 500 °C at a rate of 0.5 °C min^−1^. The BC was pyrolyzed at a constant temperature for 2 h before allowing the furnace to cool completely under a continuous nitrogen gas purge at a rate of 40 mL min^−1^. After cooling, BC samples were ground and sieved to obtain particle sizes of <0.5 mm.

To modify the surface of the BC, 0.5 g BC samples were washed with 200 mL of 0.05 M HCl for 6 h to dissolve and eliminate soluble salts and alkalis that could potentially interfere with the sorption analysis. Subsequently, the BC samples were subjected to 30 min washes using 100 mL of 1 M CaCl_2_ solution. This was followed by four washes with ultra-pure deionized water to remove the remaining excess salts. The washes were carried out in a glass beaker placed on a magnetic stirrer at 300 rpm, while vacuum filtration was performed using a 1.2 μm glass microfiber filter paper to remove the aqueous phase between each wash step. Finally, the BC samples were dried at 105 °C for 8 h after the final wash.

### 2.3. Biochar Characterization and Analysis

The pH and EC (electrical conductivity) of BC were measured at a 1:1 liquid-to-solid ratio after mixing for 30 min in deionized water [29]. The measurements were performed using the HQ440D Laboratory Multi-Parameter Meter (HACH, Loveland, CO, USA). The point of zero charge (PZC) is the pH value of a solution at which the surface density of positive charges (cation contribution) equals the surface density of negative charges (anions). The PZC analyses were performed using similar methods to those described in the literature [40]. Unmodified and modified BC samples (0.02 g) were weighed in triplicate and saturated in 10 mL solutions of 0.01 M NaCl. The pH of the solution in each vial was initially adjusted using predetermined quantities of 1 M HCl and 1 M NaOH before the addition of BC. The BC samples were shaken for 4 h, and the pH values were measured at the end of the experiment.

Scanning electron microscopy (SEM), energy-dispersive X-ray analysis (EDX), and X-ray photoelectron spectroscopy (XPS) were used to observe the morphological characteristics of the BC samples and to quantify the photoelectric activity of elements on the BC surface. The JSM-6610 LV SEM model (JEOL, Peaboy, MA, USA)was used for performing SEM and EDX observations, while the Nexsa XPS device (Thermo Fisher Scientific, Waltham, MA, USA) was used for XPS analysis. To understand how the trace metal content of the preferred BC may impact the sorption process, modified BC samples were digested, and the metal concentration was quantified using ICP-MS. The metal extraction was performed using the reverse aqua regia process [41]. A 1 g sample of BC was treated with 10 mL of an acid mixture of HNO_3_ and HCl (3:1). For digestion, the solution was ultrasonically treated for 45 min at ambient temperature. The mixture was digested for 60 min at 120 °C on a hotplate. The digested samples were then filtered and diluted to 150 mL for ICP-MS analysis in a regulated laboratory following the EPA 200.7 test methods for water samples. Each standard was digested in triplicate for each acid mixture using adequate reagent blanks. The functional groups in the infrared spectra on the surface of the biomass, unmodified, modified, and spent BC samples were recorded with Fourier-transform infrared spectroscopy (FTIR) using a Bruker ALPHA II FTIR Spectrometer. The spectra were recorded by averaging 32 scans from 4000 to 400 cm^−1^ to observe strong diamond IR absorption peaks. The spectrum peaks were studied to understand the surface functionality of analyzed samples.

### 2.4. Batch Sorption Experiments

Batch sorption experiments were conducted to determine the effect of solution pH, BC feedstock, and BC modification on NH_4_^+^ and NO_3_^−^ sorption to BC. First, 0.1 g BC was weighed into 40 mL capped glass vials in duplicate with a total of 25 mL solution containing NH_4_NO_3_ as the inorganic nitrogen source and a variable amount of Ca(OH)_2_. To make each solution, 10–20 mL of deionized water was pipetted into the vial in varying volumes, followed by 0–10 mL of standardized 1 mmol L^−1^ Ca(OH)_2_ and finally 5 mL of 3.75 mmol L^−1^ NH_4_NO_3_ solution. Adding the solutions in this order minimized the risk of NH_3_ volatilization by allowing the BC to react with the Ca(OH)_2_ before the addition of NH_4_^+.^ The vials were placed on a tube rotator at 20 rpm for 24 h. After 1 h of settling, the supernatants were analyzed for pH. They were then filtered using a 0.45 μm PTFE syringe filter membrane (Corning Inc., Corning, NY, USA). Residual NH_4_^+^, NO_3_^−^, and total inorganic nitrogen were analyzed using HACH Method 10031, Method 10206, and Method 10021, respectively. To understand the impact of the inorganic nitrogen source on the sorption, the experiments were also repeated using (NH_4_)_2_SO_4_.

The sorption capacity of the BC was calculated from the measured aqueous nitrogen concentration using Equation (1):

(1) $$A=\frac{V(C_{1}-C_{2})}{S}$$

where A = the amount of adsorbed nitrogen ion (mg g^−1^), C_1_ = the initial nitrogen ion concentration (mg L^−1^), C_2_ = the equilibrium nitrogen ion concentration (mg L^−1^), V = the volume of solution (L), and S = the biochar mass (g).

## 3. Results and Discussion

The electrical conductivity of *Pistia stratiotes* biomass measured over time showed a consistent increase in EC values. This implied that the plant material contained ions and elements that gradually leached out of the biomass, thus providing competing ions that could interfere with the sorption experiments. *Pistia stratiotes* biomass also contained a total nitrogen concentration > 25.0 mg L^−1^ N and released a variable portion of nitrogen into the test solutions during experimental processes. This observation corroborates existing literary evidence that *Pistia stratiotes* plants uptake nitrogen when growing in lakes and rivers [42,43]. Various studies have demonstrated that the sorption capacity of BC can be increased via nitrogen doping [29]; hence, to maximize and harness the potential of the *Pistia stratiotes* biomass for nitrogen removal, the material was further processed into BC, which served as a means of fixing the existing nitrogen and carbon contained in the biomass structure. Nitrogen gas used in the pyrolysis process is non-polar and inert and does not interact strongly with the BC surface or become absorbed by the material during pyrolysis. Hence, the nitrogen used during pyrolysis is not a significant source of nitrogen in BC. Upon testing for the possibility of nitrogen leaching from the BC, the total nitrogen concentration leaching from all BC samples was <0.04 mg L^−1^ N. Biomass pyrolysis yielded an average of 47% BC.

### 3.1. Biochar Characterization

#### 3.1.1. Physiochemical Characteristics

The measurement of the pH of the BC samples (Table 1) revealed a decrease after chemical modification. This can be attributed to the acid-to-solid ratio used in the modification process. The chemical modification of BC by acid washing has been proven to increase the surface area, porosity, and cation exchange capacity on the BC surface [29,44]. Several studies have measured the EC of BC produced from a variety of feedstocks, including lignin-containing biomass. These studies have recorded EC values that differ widely and range from 19.1 to 629.6 × 10^5^ µS cm^−1^, and these values increase with the pyrolysis temperature [45,46,47]. These EC values have been attributed to BC properties such as metal, oxygen, and carbon content; particle and graphitic crystal sizes; and surface area. In this study, the EC measured after pyrolysis at 500 °C was 69.67 µS cm^−1^, and after modification, this value decreased by as much as 50%. The EC value decreased by 91.2%, which implies that the trace metals contained in the BC were significantly dissolved during acid modification.

The PZC of unmodified and modified BC samples were obtained to assess the possibility of electrostatic interactions between the BC surface and inorganic nitrogen compounds. At a pH lower than that of the PZC, the material is positively charged and preferentially adsorbs anions. On the contrary, at a pH higher than the PZC of the material, the adsorption of cations is enhanced. This property can be developed to maximize the electrostatic interactions and adsorb the maximum amount of inorganic nitrogen ions on the BC surface. The PZC values for the unmodified BC and modified BC were 9 and 6, respectively (Table 1). This implies that based on the PZC values obtained, the unmodified BC would have a greater affinity for cations (e.g., NH_4_^+^), whereas the modified BC has a better affinity for anions (e.g., NO_3_^−^).

#### 3.1.2. Surface Morphology and Elemental Composition

**Figure 1:**
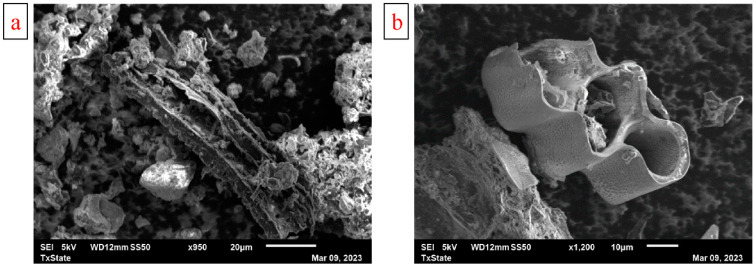
SEM Images of (**a**) unmodified BC and (**b**) and modified BC.

Surface characterization was performed for unmodified and modified BC using SEM/EDX. The analysis revealed that all the BC samples were diverse in structural composition (Figure 1). This can be attributed to the physical degradation of lignin in the biomass structure caused by milling. Different portions of the BC revealed a variance in pore size and structure. SEM micrographs revealed the porous structure of the modified BC. The modified BC appeared to have cleaner pores compared to the unmodified BC. Measurements taken from the SEM images show an abundance of macropores with pore sizes ≥ 4 µm as well as some mesoporous features observed on the BC surface. The inner surface of the macropores that feature smaller pores observed in modified BC could provide additional sorption sites beyond the available external surface [48].

The mass and atom percentages of carbon, nitrogen, and oxygen in the unmodified BC and modified BC were analyzed by EDX (Table 2). The EDX analysis also revealed the presence of other elements (Mg, Fe, K, P, Al, S, Si, Ca, Cl) on the biochar surface that may influence the sorption properties of BC. The mass and atom percentages of carbon in unmodified BC clearly show that BC is rich in carbon (i.e., >40 mass% and >50 atom%). The modification made the percentages of carbon in BC increase further, whereas it slightly decreased the percentages of oxygen. Oxygen-containing functional groups in biochar, such as carboxyl, carbonyl, phenolic hydroxyl, and lactone groups, are good adsorption indicators for cations [30]. The percentages of elements (phosphorus, magnesium, sulfur, potassium, calcium) decreased after BC modification (acid washing). However, an increase in relative mass percentage was observed for silica, chlorine, iron, and aluminum after the modification of BC. We can infer that the modification of BC did not considerably reduce the concentration of these elements on the biochar surface. In the case of silica, the increased concentration can be attributed to the insoluble nature of non-polar silica in water during the acid washing used in the modification procedure. Silica is mainly soluble in a highly alkaline condition (e.g., pH > 9) [49]. The increase in chloride on the BC surface may be related to the addition of CaCl_2_ during the modification of BC. The concentration of chlorine added due to the modification of BC was very minimal, and the Cl ions on the BC surface were completely exchanged during the sorption process to aid in NH_4_^+^ removal. Calcium, on the other hand, was reduced during the modification process but increased on the BC surface after the sorption process due to the addition of Ca(OH)_2_ to the solution for the batch experiments. The slight increase in the percentages of nitrogen after the NH_4_^+^ and NO_3_^−^ sorption study is indicative of the nitrogen sorbed by the BC.

Results from the batch experiments showed a transformation from NO_3_^−^ to NH_4_^+^ and vice versa. Therefore, to understand the effect of metals on BC and to investigate the effect of metals in the BC on N transformation, the modified BC was analyzed for metals [50,51,52]. The elements analyzed include cadmium, calcium, cobalt, copper, iron, magnesium, manganese, nickel, and zinc due to evidence from studies that these elements potentially affect nitrogen adsorption processes just as nitrogen in BC can influence metal sorption [53]. The results indicate that cadmium and cobalt were not present in the modified BC extracts, thus eliminating the possibility of interactions by these elements (Table 3). Calcium (7.85 mg L^−1^) and magnesium (5.93 mg L^−1^) were present in the samples, and the concentrations may be linked to the growing environment as well as the addition of calcium during biochar modification. This reveals the availability of calcium ions for use during the sorption of nitrogen. The presence of iron, manganese, nickel, and zinc in the BC extract may result in competition between these metals and nitrogen ions for available sorption sites. However, this may not be solely responsible for the sorption capacity of BC.

#### 3.1.3. Chemical State Identification Using XPS

**Figure 2:**
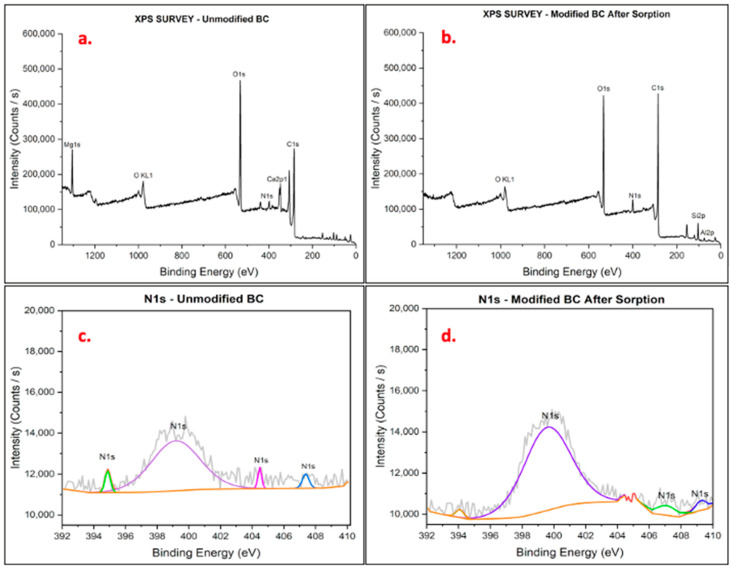
XPS survey scans of (**a**) unmodified BC and (**b**) modified BC after sorption study and deconvoluted nitrogen chemical state peaks for (**c**) unmodified BC and (**d**) modified BC after sorption study.

To identify the chemical state of the functional groups on the BC, XPS analysis was performed. The survey scan (Figure 2) showed the presence of six major peaks related to C1s, OKL1, O1s, Mg1s, N1s, and Ca2p1 in the unmodified BC. After modification, C1s and O1s peaks were the most intense, with smaller peaks for other previously detected chemical states except Mg1s. The modification also allowed for the quantification of Al and Si chemical states. A survey scan of BC after the batch experiment showed similar peaks to those seen after modification, with less intensity for all chemical states, including N1s, and a significant decrease in available carbon. These results correspond to the quantification observed from the EDX analysis. For the deconvoluted peaks, binding energy values were confirmed using the National Institute of Standards and Technology X-ray Photoelectron Spectroscopy Database (SRD 20), Version 5.0 [54]. For the N1s chemical state, one major peak at ≈399 eV was observed, with other less intense peaks between ≈394 and 409 eV. Before the modification of BC, more minor peaks were observed, which disappeared after modification, and an extra peak reappeared at ≈409 eV after sorption. For the O1s chemical state, all stages of BC analyzed showed one major peak at ≈531.68 eV, with a reduction in intensity after the modification and batch experiment, respectively. C1s was observed at ≈284 eV for all BC samples analyzed. Peak intensity increased after modification but decreased after sorption. However, the intensity of C1s in the modified BC before and after sorption exceeded the peak intensity of the unmodified BC. For the Cl2p chemical state, one main peak was observed at ≈195 eV, and there were three other less intense peaks. After modification, the peak distribution increased, and a shift in the binding energy of the initial peaks was observed. After sorption, five peaks with similar binding energy to the peaks after modification but with less intensity were observed on the BC surface. Before modification and after modification, Ca2p peaks were observed at ≈347 and 351 eV. However, the intensity of peaks after modification was reduced significantly. After sorption, an additional peak was observed at ≈345 eV, but all peaks had comparable intensity to the peaks after modification.

#### 3.1.4. Surface Functional Groups

**Figure 3:**
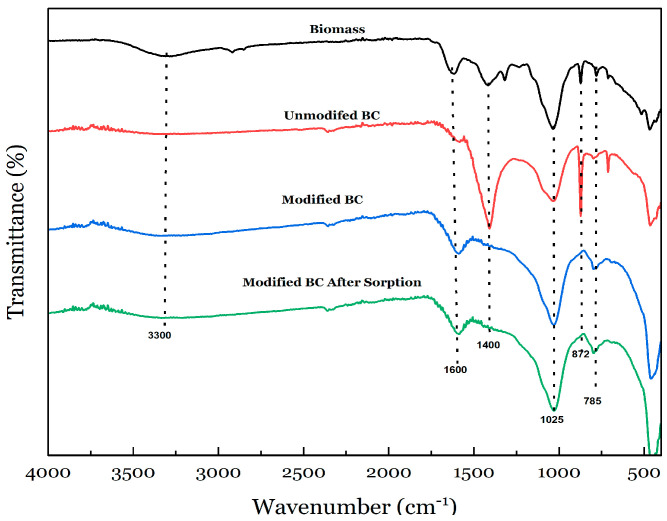
FTIR spectra of *Pistia stratiotes* biomass, unmodified BC, modified BC, and modified BC after sorption experiments.

The BC samples analyzed for the FTIR spectra (Figure 3) show the progression of *Pistia stratiotes* surface functional groups from the biomass stage to after the sorption tests. The most dramatic change is the loss of peaks that occurs as the material changes from biomass to BC and the loss of peaks due to the modification process. The observation of peak changes indicates the presence of a peak at ≈3300 cm^−1^ in the biomass, which decreases in intensity after pyrolysis and strengthens with a weaker transmittance after the modification of the BC and stays the same after the sorption experiments. Typically, the characteristic peak around ≈3300 cm^−1^ corresponds to the possibility of oxygen-related bonding formed on the surface of the biomass material. The narrow bands between 3000 cm^−1^ and 2000 cm^−1^ are attributed to C-C absorption bonds, while these could also indicate the presence of C≡C bonds. The weak/moderate band around 1600 cm^−1^ was mainly assigned to the stretching vibration of O–H and to C=O stretching vibrations. Another noticeable difference is the peak formation at ≈1400 cm^−1^ after the pyrolysis of the biomass. The absorbance band near 1025 cm^−1^ is the C–O bending vibration, and the sharp peaks at about 785 cm^−1^ are related to the aromatic compounds and N-H groups. After pyrolysis, an out-of-plane N-H bend is observable at ≈730 cm^−1^, and a C-N aromatic stretch is observable at ≈1400 cm^−1^. Nitrogen can create a single, double, or triple bond with carbon. It would be ideal if peaks from one or more of these functional groups could indicate the presence of organic nitrogen in a sample, but this is not the case. Instead, N-H stretching peaks are the most reliable predictor of nitrogen in a sample.

Another noticeable change is the reduction in carbon and oxygen functional peaks, as well as a shift in the position of oxidized nitrogen peaks, which points to the possible uptake of nitrate by the BC. The series of spectra also suggests a loss of lignocellulosic functional groups. The peak intensity increases at ≈875 cm^−1^ when the material is converted from biomass to BC; however, the peak disappears after the modification of the BC sample. Although the peak positions and intensity stay relatively the same after modification and after the sorption experiment, there is a slight reduction in peak intensity at ≈471 cm^−1^. This same peak was also observed to have increased in intensity after the modification of BC. The BC preparation method and temperature—slow pyrolysis, 500 °C—could be responsible for fewer oxygen-containing functional groups, especially the strong O-H stretch around ≈3400 cm^−1^, the carboxylic C stretch functional groups around ≈1700 cm^−1^, and a stronger aromatic C-H stretch signal at ≈3050 cm^−1^. These groups play an important role in increasing the efficiency of the N sorption process. Hence, the absence of these groups on the BC surface could explain the results generated from the sorption experiments.

### 3.2. Batch Sorption Tests and Effect of Reaction pH

Batch experiments were conducted to investigate the sorption of NH_4_^+^ and NO_3_^−^ by unmodified and modified BC samples. The experiments examined the response of the BC when the pH was acidic, alkali, and near neutral in the batch system, following evidence from the literature that NH_4_^+^ sorption is better at a neutral pH and NO_3_^−^ at an acidic pH [10,12]. Observations from the batch studies also showed that the pH of the BC surface as influenced by the modification process was very important for dictating the pH of the system. The addition of Ca(OH)_2_, which is a basic compound, had a notable but minimal effect on the pH of the solution in the sorption experiments. For instance, in cases where the BC surface was very acidic, the overall solution pH was acidic; thus, the addition of Ca(OH)_2_ had only subtle effects on swaying the reaction pH. To confirm this, the pH of the solution used in the reaction with the exclusion of the BC was measured. The pH varied as expected depending on the concentration of Ca(OH)_2_ included. The pH measurements were performed at the beginning of the experiment and after 24 h. Using the measured values of solution pH, it was possible to infer which nitrogen compound would be better reduced in the experiment.

**Figure 4:**
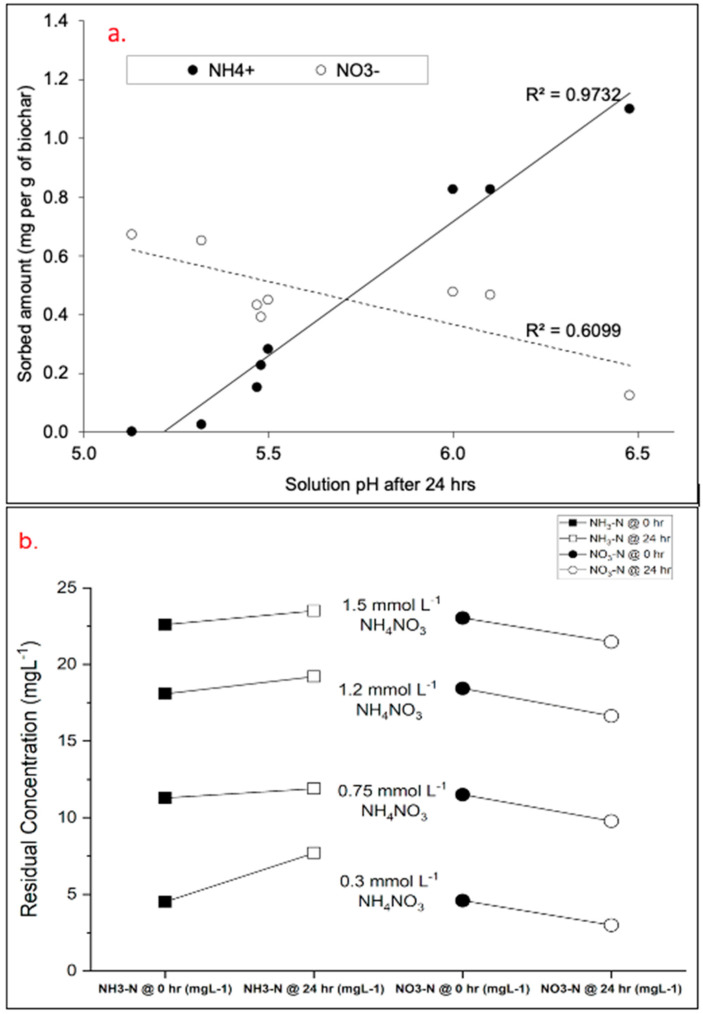
(**a**) Sorbed amount of NH_4_^+^ and NO_3_^−^ on modified BC after 24 h at different reaction pH values and (**b**) concentration of NH_4_^+^ and NO_3_^−^ before and after adsorption when NH_4_NO_3_ concentration is varied.

The results showed that the presence of unmodified BC acted more as a transformation agent due to the observed shift in the concentration of NH_4_^+^ and NO_3_^−^ and vice versa. In the case where the NH_4_^+^ concentration was reduced after 24 h, the concentration of NO_3_^−^ was observed to increase. As shown in Figure 4a, the modified BC performed better for NH_4_^+^ sorption at a near-neutral pH, showing >1.0 mg NH_4_^+^ sorbed per g of modified BC and <0.2 mg NO_3_^−^ sorbed per g of modified BC. The opposite phenomenon was observed at the lowest pH (~5.1), where >0.6 mg NO_3_^−^ was sorbed per g of modified BC while NH_4_^+^ sorption was negligible. Furthermore, the measurement of total inorganic nitrogen at 0 and 24 h showed that the inorganic nitrogen concentration in the solution remained unchanged. When the modified BC was used in adsorption tests, there was a notable reduction in the concentration of NO_3_^−^ without the formation of new NH_4_^+^ when the solution was acidic, and at near-neutral pH values, the adsorption of NH_4_^+^ occurred without the formation of NO_3_^−^. The adsorption of the nitrogen compounds from the aqueous solution was confirmed by the reduction in total inorganic concentrations measured after 24 h.

The result of the control experiments showed that the addition of Ca(OH)_2_ was crucial to enhancing the adsorption of NH_4_^+^ and NO_3_^−^. Despite achieving a considerably higher NO_3_^−^ and NH_4_^+^ reduction using modified BC at pH values < 5.4 and >7.4, respectively, the exclusion of Ca(OH)_2_ in the solution resulted in nearly negligible NH_4_^+^ sorption for all BC samples (<0.01 mg g^−1^). When Ca(OH)_2_ was excluded from the solution, total inorganic nitrogen concentrations remained unchanged after 24 h.

Across all batch systems where the Ca(OH)_2_ concentration was varied, an overall trend was observed concerning the influence of pH on NH_4_^+^ and NO_3_^−^ concentrations at the end of the reaction. When pH < 7, a reduction was observed in NO_3_^−^ values. This occurred mainly when modified BC was combined with a lower volume of Ca(OH)_2_. When unmodified BC was used with the addition of Ca(OH)_2_, the pH of the system ranged from 8.13 to 10.17. When the pH was less than 8.5, a reduction in NH_4_^+^ and an increase in NO_3_^−^ was recorded. For pH values exceeding 8.5, the concentration of NH_4_^+^ and NO_3_^−^ remained unchanged in the system. Summarily, it was observed that solutions containing unmodified BC where NH_4_^+^ concentration was reduced, NO_3_^−^ concentration increased, and vice versa.

When the concentration of NH_4_NO_3_ was varied and the concentration of Ca(OH)_2_ was fixed, the reaction pH remained acidic (5.9–6.1). Although a reduction in the NO_3_^−^ concentration was observed across all systems, an increase in the NH_4_^+^ concentration was recorded (Figure 4b). This likely indicates that the NO_3_^−^ in the system was not adsorbed by the modified BC. This phenomenon led to the assumption that a transformative reaction was occurring in some of the batch systems rather than a sorption process. However, the effect of the solution pH due to Ca(OH)_2_ addition and the BC pH was instrumental in determining which nitrogen compound had a higher concentration in the system. At an acidic pH of 4.8–6.5, the NO_3_^−^ concentration was considerably reduced (20%) in the system. The addition of Ca(OH)_2_ was intended to vary the pH of the test systems; however, the changes observed with the increasing volume of the compound contributed minimal changes to the system pH after 24 h.

### 3.3. Potential Oxidative Transformation of NH_4_^+^ to NO_3_^−^

To corroborate the findings from the sorption study with NH_4_NO_3_ as the NH_4_^+^ and NO_3_^−^ sources, (NH_4_)_2_SO_4_ and KNO_3_ were used as different NH_4_^+^ and NO_3_^−^ sources, respectively, in an additional sorption study with modified BC only. In this additional sorption study with (NH_4_)_2_SO_4_, the NH_4_^+^ concentration was decreased from 10 to 8.9 mg L^−1^ NH_3_^+^-N, but the NO_3_^−^ concentration was increased from 0 to 2.6 mg L^−1^ NO_3_^−^-N. The final pH after the 24 h sorption study was 5.33, and the total inorganic nitrogen concentration in the liquid phase was maintained constant. Previously, it was found that the BC did not release any inorganic nitrogen to the liquid phase in a control reactor where BC was shaken in DI water for 24 h and the liquid phase was analyzed for NH_4_^+^, NO_3_^−^, and total inorganic nitrogen concentrations. In the additional sorption study with KNO_3_, the NO_3_^−^ concentration was decreased from 15.6 to 8.8 mg L^−1^ NO_3_^−^-N. The final pH after the 24 h sorption study was 5.79, and no NH_4_^+^ was quantified in the liquid phase. In summary, the analysis of total inorganic nitrogen and subsequent investigations shed light on the behavior of nitrogen compounds during the adsorption process using modified BC. The presence of modified BC influenced the formation of NO_3_^−^ and the reduction in NH_4_^+^, suggesting the involvement of complex oxidation reactions. Therefore, the adsorbed amount of NH_4_^+^ shown in Figure 4a, which was calculated from Eq. 1, may not have been solely attributed to BC adsorption. Rather, it may have been attributed to the transformation of NH_4_^+^ to NO_3_^−^ by modified BC. According to Liao et al. [55], oxygen-centered free radicals are ubiquitously present in BC and induce strong hydroxyl radicals in the aqueous phase. Fang et al. [56] reported the formation of persistent free radicals in BC and utilized them to produce sulfate radicals in the degradation of polychlorinated biphenyls. Understanding these mechanisms and exploring modification methods can optimize the adsorption capabilities of *Pistia stratiotes* BC and provide avenues for sustainable nitrogen recovery and utilization. Further studies are warranted to explore the unique oxidative reactivity of BC in contaminant reduction and to advance oxidative remediation technologies for contaminated sites.

Additionally, the results of EDX and FTIR analyses of the samples reveal the possibility of nitrogen saturation in the BC between pyrolysis and modification. From this, we can infer that there is a need for a BC modification method that displaces nitrogen ions present on the BC to increase the available surface area for nitrogen adsorption. Alternatively, biomass can be pretreated before pyrolysis to alter the functional groups on the BC surface, thus enhancing nitrogen sorption. Another option would be to optimize the BC for the removal of target contaminants like metals since nitrogen-doped biochar has proven effective for metal sorption. BC may also be prepared for agricultural applications for the slow release of nutrients for plant growth.

## 4. Conclusions

Based on the experimental results from the sorptive removal of aqueous NH_4_^+^ and NO_3_^−^ by *Pistia stratiotes*-derived biochar, the following conclusions are derived:

- The acid modification of biochar reduced the pH and electrical conductivity, making modified biochar more agreeable for environmental applications. It also decreased the point of zero charge, making it more plausible for NO_3_^−^ adsorption.
- SEM revealed that the modified biochar had a smoother and more hollow porous structure than that unmodified biochar. Energy-dispersive X-ray analysis showed that modified biochar had higher percentages of carbon and nitrogen but a lower percentage of oxygen.
- Modified biochar performed better for NH_4_^+^ sorption at a near-neutral pH, showing >1.0 mg NH_4_^+^ sorbed per g of modified biochar and <0.2 mg NO_3_^−^ sorbed per g of modified biochar. The opposite phenomenon was observed at the lowest pH (~5.1), where >0.6 mg NO_3_^−^ was sorbed per g of modified biochar, while NH_4_^+^ sorption was negligible.
- However, the calculated sorbed amount of NH_4_^+^ based on the decreased NH_4_+ in the aqueous phase may not be solely ascribed to the sorption of NH_4_^+^ by biochar. It could have been associated with the oxidative transformation of NH_4_^+^ to NO_3_^−^ by biochar.
- Additionally, the reduction in NO_3_^−^ recorded when the concentration of NH_4_NO_3_ was varied may not be directly related to adsorption by modified biochar but rather to the transformation of NO_3_^−^ to NH_4_^+^.

Overall, the findings from this study imply an alternative way to reduce inorganic nitrogen compounds in water with a more sustainable technique utilizing biochar derived from the invasive aquatic plant *Pistia stratiotes* L.

## Figures and Tables

**Table 1:** Physicochemical characteristics of the unmodified and modified BC from *Pistia stratiotes* L.

	EC (µS/cm)	pH in Deionized Water	PZC	Total N (mg L^−1^ N)
Unmodified BC	69.97	10.08	9.0	0.04
Modified BC	6.19	4.92	6.0	0.0

**Table 2:** Mass and atomic percentages concentration of elements on the surface of unmodified BC, modified BC, and modified BC after the sorption study.

Element	Unmodified BC		Modified BC before Sorption		Modified BC after Sorption	
	Mass%	Atom%	Mass%	Atom%	Mass%	Atom%
C	41.43	50.25	49.30	56.70	48.86	56.23
N	12.47	12.97	15.05	14.84	16.02	15.81
O	34.61	31.51	29.59	25.55	28.88	24.95
Mg	2.31	1.38	0.72	0.41	0.87	0.50
Al	0.73	0.39	0.91	0.47	0.90	0.46
Si	2.64	1.37	3.50	1.72	3.52	1.73
P	0.18	0.09	0.06	0.02	0.07	0.03
S	0.11	0.05	0.07	0.03	0.09	0.04
Cl	0.09	0.04	0.11	0.04	*n.d	*n.d
K	0.42	0.16	0.21	0.08	0.21	0.08
Ca	4.78	1.73	0.22	0.08	0.30	0.10
Fe	0.23	0.06	0.26	0.06	0.28	0.07
Total	100	100	100	100	100	100

*n.d—not detected.

**Table 3:** Mean metal concentrations in biochar analyzed using ICP-MS.

Test Method: EPA 200.7		
Element	Concentration per g of BC (mg L^−1^)	Practical Quantitation Limit
Cadmium	<0.005	0.005
Calcium	7.850	1.000
Cobalt	<0.010	0.010
Copper	0.027	0.010
Iron	4.280	0.050
Magnesium	5.930	0.050
Manganese	0.270	0.010
Nickel	0.034	0.010
Zinc	0.156	0.010

## Data Availability

Data are contained within the article.
